# Lower doses of carvedilol in Japanese heart failure patients with reduced ejection fraction could show the potential to be non-inferior to higher doses in US patients: An international collaborative observational study

**DOI:** 10.1371/journal.pone.0299510

**Published:** 2024-03-07

**Authors:** Makiko Maeda, Douglas Humber, Eisuke Hida, Tomohito Ohtani, Guannan Wang, Tong Wu, Shiori Takeda, Jacinta N. Situ, Jun Hayashi, Shinpei Nonen, Toshihiro Takeda, Hiroshi Okamoto, Masatsugu Hori, Yasushi Sakata, Yasushi Fujio, Shirley M. Tsunoda

**Affiliations:** 1 Laboratory of Clinical Pharmacology, Department of Pharmaceutical Sciences, Osaka University, Osaka, Japan; 2 Medical Center for Translational Research, Osaka University Hospital, Osaka, Japan; 3 Department of Molecular Pharmaceutical Science, Graduate School of Medicine, Osaka University, Osaka, Japan; 4 Skaggs School of Pharmacy and Pharmaceutical Sciences, University of California San Diego, San Diego, CA, United States of America; 5 San Diego Health, University of California San Diego, San Diego, CA, United States of America; 6 Department of Biostatistics and Data Science, Osaka University Graduate School of Medicine, Osaka University, Osaka, Japan; 7 Department of Cardiovascular Medicine, Graduate School of Medicine, Osaka University, Osaka, Japan; 8 School of Pharmacy, Hyogo Medical University, Hyogo, Japan; 9 Department of Medical Informatics, Osaka University Graduate School of Medicine, Osaka University, Osaka, Japan; 10 Aishin-Memorial Hospital, Sapporo, Japan; 11 National Hospital Organization Osaka National Hospital, Osaka, Japan; 12 Laboratory of Clinical Science and Biomedicine, Graduate School of Pharmaceutical Sciences, Osaka University, Osaka, Japan; 13 Integrated Frontier Research for Medical Science Division, Institute for Open and Transdisciplinary Research Initiatives, Osaka University, Osaka, Japan; Tehran University of Medical Sciences, ISLAMIC REPUBLIC OF IRAN

## Abstract

The Japanese national guidelines recommend significantly lower doses of carvedilol for heart failure with reduced ejection fraction (HFrEF) management than the US guidelines. Using real-world data, we determined whether initial and target doses of carvedilol in Japanese patients (JPNs) differ from those in US patients (USPs), especially in Asian Americans (ASA) and Caucasians (CA), and investigated differences in outcomes. We collected data from the electronic medical records, including demographics, carvedilol dosing, tolerability, cardiac functional indicators like EF, cardiovascular events including all-cause deaths, and laboratory values from the University of California, San Diego Health and Osaka University. JPNs had significantly lower doses (mg/day) of carvedilol initiation (66 USPs composed of 38 CAs and 28 ASAs, 17.1±16.2; 93 JPNs, 4.3±4.2, *p*<0.001) and one year after initiation (33.0±21.8; 11.2±6.5, *p*<0.001), and a significantly lower relative rate (RR) of dose discontinuation and reduction than USPs (RR: 0.406, 95% confidence interval (CI): 0.181–0.911, *p*<0.05). CAs showed the highest reduction rate (0.184), and ASAs had the highest discontinuation rate (0.107). A slight mean difference with narrow 95% CI ranges straddling zero was observed between the two regions in the change from the baseline of each cardiac functional indicator (LVEF, -0.68 [−5.49–4.12]; LVDd, −0.55 [−3.24–2.15]; LVDd index, −0.25 [−1.92–1.43]; LVDs, −0.03 [−3.84–3.90]; LVDs index, −0.04 [−2.38–2.30]; heart rate, 1.62 [−3.07–6.32]). The event-free survival showed no difference (*p* = 0.172) among the races. Conclusively, despite JPNs exhibiting markedly lower carvedilol doses, their dose effectiveness has the potential to be non-inferior to that in USPs. Dose de-escalation, not discontinuation, could be an option in some Asian and ASA HFrEF patients intolerable to high doses of carvedilol.

## Introduction

Heart failure (HF) is one of the leading causes of death in the United States of America (US) [1] and Japan [2]. According to the evidence of several well-designed randomized controlled trials (RCTs) for patients with HF with reduced ejection fraction (HFrEF), the HF guidelines strongly recommend the use of combination therapy with foundational drugs, such as angiotensin-converting-enzyme inhibitors or angiotensin-receptor blockers, β-blockers, mineralocorticoid receptor antagonists, angiotensin-receptor neprilysin inhibitors (ARNIs), and sodium-glucose co-transporter 2 (SGLT2) inhibitors [3, 4]. ß-blockers such as bisoprolol [5, 6], metoprolol succinate [7, 8], and carvedilol [9–11] have demonstrated an improvement in cardiac mortality and morbidity by more than 30% compared to placebo and have formed a cornerstone for the therapeutic management of HFrEF [3, 4].

HF management proposed by the respective guidelines of the American College of Cardiology (ACC)/American Heart Association (AHA), European Society of Cardiology (ESC) [3], and the Japanese Circulation Society (JCS) [4] are similar. These guidelines recommend initiating with the lowest possible dose of available agents and then gradually titrating to maximally tolerated doses, targeting the doses shown in the landmark clinical trials to be ideal. The recommended initial and target doses of approved β-blockers for HFrEF treatment also significantly differ between ACC/AHA and JCS because each national guideline has been developed with evidence from the dominant ethnic group in the country (S1 Table). Among these ß-blockers, carvedilol is approved in both the US and Japan and is more prescribed than bisoprolol, especially in the US. The MOCHA study, with about 80% of enrolled participants being Caucasian, showed an association of carvedilol with dose-related improvements (12.5, 25, 50 mg/day) in LV function and survival in HFrEF [9]. The trials performed with Japanese HFrEF patients showed that lower doses, such as 5 mg/day, reduced morbidity and mortality by 71% compared to placebo [12] with no significant dose-related improvement (2.5, 5, 20 mg/day) in all-cause mortality [13].

According to these pieces of evidence, the established target daily dose in the USA and Europe is more than twice that in Japan. However, it is unclear whether carvedilol doses lower than 20 mg/day in Japanese HFrEF patients could lead to less effectiveness compared to carvedilol doses higher than 20 mg/day, which may be used for Asian Americans with HFrEF according to the US guidelines. Simultaneously, higher doses of carvedilol for Asian Americans with HFrEF could lead to more frequent side effects or intolerability.

Given this background, we used real-world data to compare the dose difference among Japanese and US patients, especially Asian Americans and Caucasians, with HFrEF, who were expected to follow each national guideline recommendation. Then, we analyzed how differences in carvedilol doses affect outcomes and tolerability among the races in the two regions.

## Materials and methods

### Study design, ethics, and subjects

This was an international, collaborative, observational study conducted at the University of California, San Diego Health (UCSDH) and the Skaggs School of Pharmacy and Pharmaceutical Sciences in the USA, the Osaka University Hospital (OUH), and the Department of Pharmaceutical Sciences of Osaka University (OU) in Japan. Each local ethics committee approved the study protocol at UCSD (Project #: 190590X), OUH (Approval #; 19107), and the Department of Pharmaceutical Sciences of OU (Approval #; YAKU-HITO 2019–4). Then, it was performed under The Code of Ethics of the 1964 Declaration of Helsinki and its later amendments. A waiver of informed consent from each patient was granted by the institutional review boards as the study involved no more than minimal risk and met the conditions of the ethical guidelines for medical and health research involving human subjects. The study subjects were self-reported Asian Americans (ASAs) and Caucasians (CAs) treated at UCSDH from 2003 to 2019, and Japanese (JPNs) treated at OUH from 2008 to 2019, and followed for one year after the initiation of carvedilol at each hospital. Considering the feasibility of data collection, all the clinical data were collected at an independent core lab of universities using the EPIC system at UCSDH from 17 June 2019 to 14 May 2022 and the electronic patient record (EPR) system at the OUH from 22 July 2019 to 24 February 2022 by the independent individuals from the researchers involved in data analysis. Data collected within EPIC and the EPR system at the OUH include medication administration, demographics, procedure orders, cardiovascular conditions, and laboratory values with the examined date and time. When available, we recorded the prescribers’ rationale for the lack of up-titration to target doses or the reason for carvedilol discontinuation. Eligible study subjects were selected according to the inclusion and exclusion criteria. Researchers involved in data collection had access to information that could identify individual participants during data collection. However, after data collection, the identifiable information was anonymized at each institution, and then the data were combined for analysis.

### Inclusion and exclusion criteria

The inclusion criteria are as follows: 1) races based on the self-reported information in the medical record were ASA and CA at UCSDH and JPN at OUH; 2) age ≥ 20 years; 3) diagnosed with chronic heart failure with reduced left ventricular ejection fraction of less than 40% (HFrEF); 4) treated with carvedilol for more than four weeks after the initiation. Patients who had one or more of the following criteria were excluded: 1) no LVEF data before carvedilol initiation; 2) concurrent administration of α-blocker, β-blockers other than carvedilol and inotropic agents other than digitalis; 3) treatments only for acute HF; 4) severe aortic or mitral valve regurgitations; aortic or mitral stenosis; 5) hypertrophic obstructive cardiomyopathy; 6) cardiogenic shock; 7) grade II or III atrioventricular block; 8) unstable or resting angina; 9) any devices and surgical/non-surgical procedures to support heart function such as implantation of ventricular assist device, heart transplantation, and regenerative therapy, ablation, percutaneous coronary intervention, coronary artery bypass graft, transluminal angioplasty, and intra-aortic balloon pump support within six months before the initiation of carvedilol or during the carvedilol treatment. We also excluded patients lost to follow-up one year after carvedilol initiation.

### Primary and secondary outcomes

The primary outcome was the difference in carvedilol doses at the following three distinct points between JPNs and USPs composed of CAs and ASAs: at the carvedilol initiation for HFrEF treatment (initial), after a one-year follow-up period (final), and at the maximum doses during the period (maximum).

The secondary outcomes were tolerability to dose titration for the follow-up period and clinical effectiveness of carvedilol treatments. To assess tolerability, we compared the rates of patients who discontinued carvedilol treatment and lowered the dose of carvedilol during or after titration. To assess the clinical effectiveness, we probed the mean difference and the 95% confidential intervals (CI) for each change in cardiac functional parameters, like LVEF, left ventricular end-diastolic dimension (LVDd), LVDd index, left ventricular end-systolic dimension (LVDs), LVDs index, and heart rate. The proportion of the patients whose LVEF recovered to more than 40% and the event-free survival were also analyzed.

### Study size justification

The study subjects were the patients who fulfilled the inclusion and exclusion criteria. Referring to the results of the American Carvedilol HF Study Group [11] and the Japanese guideline-recommended dose, we conservatively assumed maximum doses of 45 ± 25 mg/day for USPs and 20 ± 25 mg/day for JPNs. With the same standard deviation (SD) value of the American Carvedilol study [11], an α error of 0.025 (two-sided) for multiplicity adjustment, and a power of 90%, the number of patients was calculated to be at least 26 subjects per group for independent two-group comparison. To effectively compare the differences in cardiac functional parameters between the two regions, taking feasibility into account, we decided to collect as many subjects as possible. The sample size calculation was performed with EZR (Saitama Medical Center, Jichi Medical University, Saitama, Japan) based on R (The R Foundation for Statistical Computing, Vienna, Austria) and R commander version 2.8–0.

### Statistical analysis

The summary statistics were reported using mean with SD and medians with interquartile range (IQR) for continuous variables. Categorical variables are reported using numbers with proportions. For the comparison among groups, analysis of variance (ANOVA) or Kruskal–Wallis analysis was used for continuous variables. As the need arose, analysis of covariance (ANCOVA) was performed with adjustment for baseline data. The chi-square test or Fisher’s exact test was performed for categorical variables. Events were defined as worsening required hospitalization for cardiac events and all deaths for one year after carvedilol initiation. Event-free survival curves for each group were estimated using the Kaplan-Meier method, and the difference was analyzed among the groups using the log-rank test.

Eligible patients were classified into three groups; “tolerated,” “discontinued,” and “lowered dose.” The patients who continued the carvedilol treatment without discontinuing or lowering the dose of carvedilol were grouped in the “tolerated” even if they did not achieve the maximal dose of 20 mg/day for JPNs and 50 mg/day for ASAs and CAs in each guideline-directed medical therapy (GDMT). The patients who discontinued the carvedilol treatment due to any adverse events or death were classified as “discontinued.” The patients who experienced lowering the dose during the titration or maintenance phase for the one-year follow-up period were in the “lowered dose,” Each patient’s highest dose during the carvedilol-treatment period was defined as the maximal dose for the patient. Each patient’s final dose was the last dose prescribed in the one-year follow-up period. The primary analysis population was defined as all subjects for whom dose data were available. Missing values of the dose were imputed with the last observation carried forward but not for the other variables.

All statistical analyses were performed at the 5% two-sided significance level using SPSS version 28 (IBM Corp) or R version 4.3.0.

## Results

### Distribution of enrolled patients

**Fig 1:**
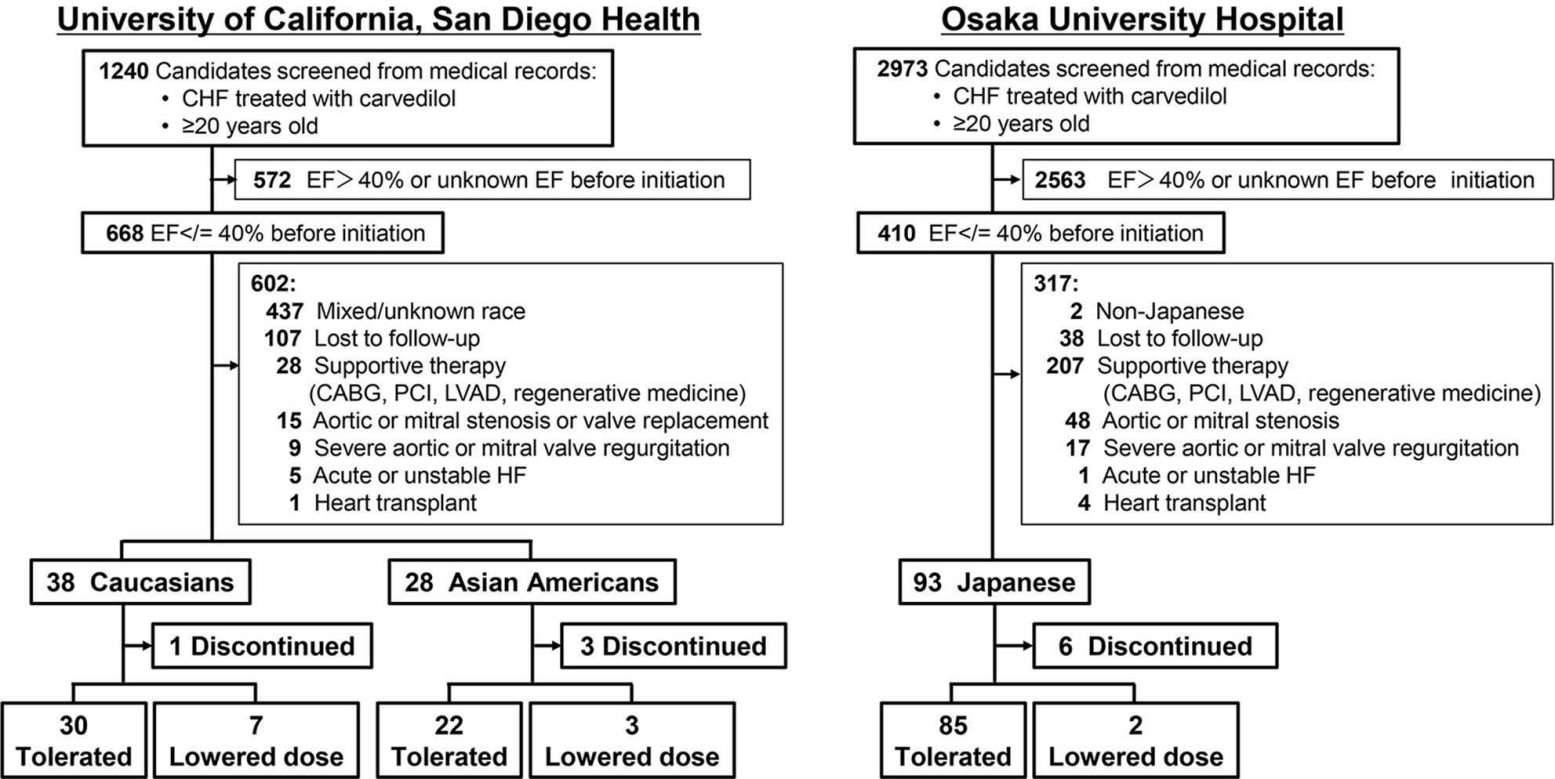
Study enrollment flow of eligible patients. UCSDH, University of California, San Diego Health; OUH, Osaka University Hospital; EF, ejection fraction; “discontinued” was defined as the discontinuation of carvedilol treatment due to any adverse event or death; “tolerated” was defined as tolerance to carvedilol treatment during the one-year follow-up period without dose discontinuation or lowering; “lowered dose” was defined as the requirement of lower doses due to any adverse event caused by carvedilol during the one-year follow-up period, but the patients could continue carvedilol treatment in the one-year follow-up.

As shown in Fig 1, 668 USPs composed of CAs and ASAs from the UCSDH and 410 JPNs from the OUH were selected as chronic HF patients with LVEF </ = 40% before carvedilol initiation. From this population, 38 CAs, 28 ASAs, and 93 JPNs met all the study criteria.

Table 1, summarizing the patients’ demographics and baseline characteristics, showed that the JPNs had significantly lower weight, height, and BMI than ASAs and CAs. There was no significant difference in other characteristics, such as age, gender, smoking status, NYHA classification, and rhythm condition among the groups. USPs, composed of CAs and ASAs, exhibited trends in demographics and baseline characteristics similar to those observed in CAs and ASAs (S2 Table).

**Table 1:** Demographics and baseline characteristics of included patients.

	Caucasian	Asian American	Japanese	*p*-value
	(n = 38)	(n = 28)	(n = 93)	
**Age (years)**	61.3 (14.6)	55.4 (20.9)	58.5 (13.4)	0.476
	58.0 (51.8–73.3)	61.0 (36.3–74.5)	61.0 (47.0–69.0)	
	38	28	93	
**Weight (kg)**	77.4 (19.6) †	74.3 (18.9) †	61.6 (13.2)	<0.001
	76.7 (65.9–86.7)	71.0 (57.4–88.8)	60.9 (51.8–68.7)	
	38	28	93	
**Height (cm)**	172.1 (9.4) *	165.6 (8.6)	163.8 (8.8)	0.001
	172.7 (162.6–178.4)	167.6 (160.7–172.7)	165.0 (159.7–169.1)	
	34	28	93	
BMI (kg/m^2^)	26.1 (4.5) *	26.8 (5.7) *	22.8 (3.8)	<0.001
	25.9 (23.5–28.7)	25.6 (22.5–31.7)	23.0 (20.3–24.7)	
	34	28	93	
**Sex**				
**Man**	26 (68.4)	19 (67.9)	65 (69.9)	0.973
**Women**	12 (31.6)	9 (32.1)	28 (30.1)	
**Smoking**				
**Never**	17 (47.2)	16 (66.7)	51 (56.0)	0.543
**Current**	8 (22.2)	2 (8.3)	18 (19.8)	
**Past**	11 (30.6)	6 (25.0)	22 (24.2)	
**NYHA class**				
**Ⅰ**	4 (20.0)	2 (10.5)	17 (21.0)	0.710
**Ⅱ/Ⅲ**	15 (75.0)	17 (89.5)	61 (75.3)	
**Ⅳ**	1 (5.0)	0 (0.0)	3 (3.7)	
**Heart rhythm**				
**Sinus**	29 (80.6)	24 (88.9)	83 (93.3)	0.111
**AF**	7 (19.4)	3 (11.1)	6 (6.7)	
**SBP (mmHg)**	120.8 (21.9)	122.6 (17.8)	117.3 (19.5)	0.416
	120.0 (103.0–138.0)	125.0 (111.1–136.3)	115.0 (104.0–128.5)	
	33	26	91	
**DBP (mmHg)**	73.2 (16.5)	74.5 (12.6)	68.6 (13.2)	0.081
	70.0 (66.0–81.0)	76.5 (64.8–82.3)	69.3 (57.3–76.3)	
	33	26	91	
eGFR (ml/min/1.73m^2^)				
**>50**	18 (90.0)	19 (86.4)	72 (77.4)	0.446
**≤50**	2 (10.0)	3 (13.6)	21 (22.6)	
**Medications**				
**ACEi / ARB**	28 (73.7)	25 (89.3)	75 (80.6)	0.286
**Diuretics**	28 (73.7)	18 (64.3)	63 (67.7)	0.695
**MRA**	15 (39.5)	8 (28.6)	29 (31.2)	0.575
**Digoxin**	10 (26.3)	3 (10.7)	7 (7.5)	0.017

BMI: body mass index; NYHA: New York Heart Association; AF: Atrial fibrillation; SBP: Systolic blood pressure; DBP: Diastolic blood pressure; eGFR: estimated glomerular filtration rate; ACEi: angiotensin-converting-enzyme inhibitor; ARB: angiotensin-receptor blocker; MRA: mineralocorticoid receptor antagonist.

Each continuous variable, such as age, weight, height, BMI, SBP, and DBP, is presented as the mean (with the SD) on the top side, the median (with the IQR) in the middle, and the number of patients at the bottom. Other variables were presented as numbers of patients with percentages of the total (%).

*, *p* < 0.001 compared with Japanese by the Kruskal–Wallis and Wilcoxon rank-sum tests;

†, *p* < 0.05 compared with Japanese by the ANOVA and Turkey-Karmer tests.

Table 2 displays the numbers of patients classified as “discontinued” and “lowered dose,” along with the frequencies within each race group. JPNs exhibited a significantly lower discontinuation and dose reduction rate than USPs, composed of CAs and ASAs, with a relative rate of 0.406 (0.086 vs. 0.212, 95% CI: 0.181 to 0.911, *p* = 0.035).

**Table 2:** Frequencies and reasons for discontinuation and dose lowering.

	Caucasian (38)	Asian American (28)	Japanese (93)
**Discontinued**	2.6% (1); Hypotension	10.7% (3); not specified	6.5% (6); Dizziness (1) Death (1) due to interstitial pneumonia Tachycardia (1) Alopecia areata (1) Numbness (1) Tachyarrhythmia (1)
**Lowered dose**	18.4% (7); not specified	10.7% (3); not specified	2.2% (2); Bradycardia (1) Hypertensive heart disease (1)

The numbers in parentheses indicate the number of patients who discontinued carvedilol or lowered the dose.

### Dose differences

**Fig 2:**
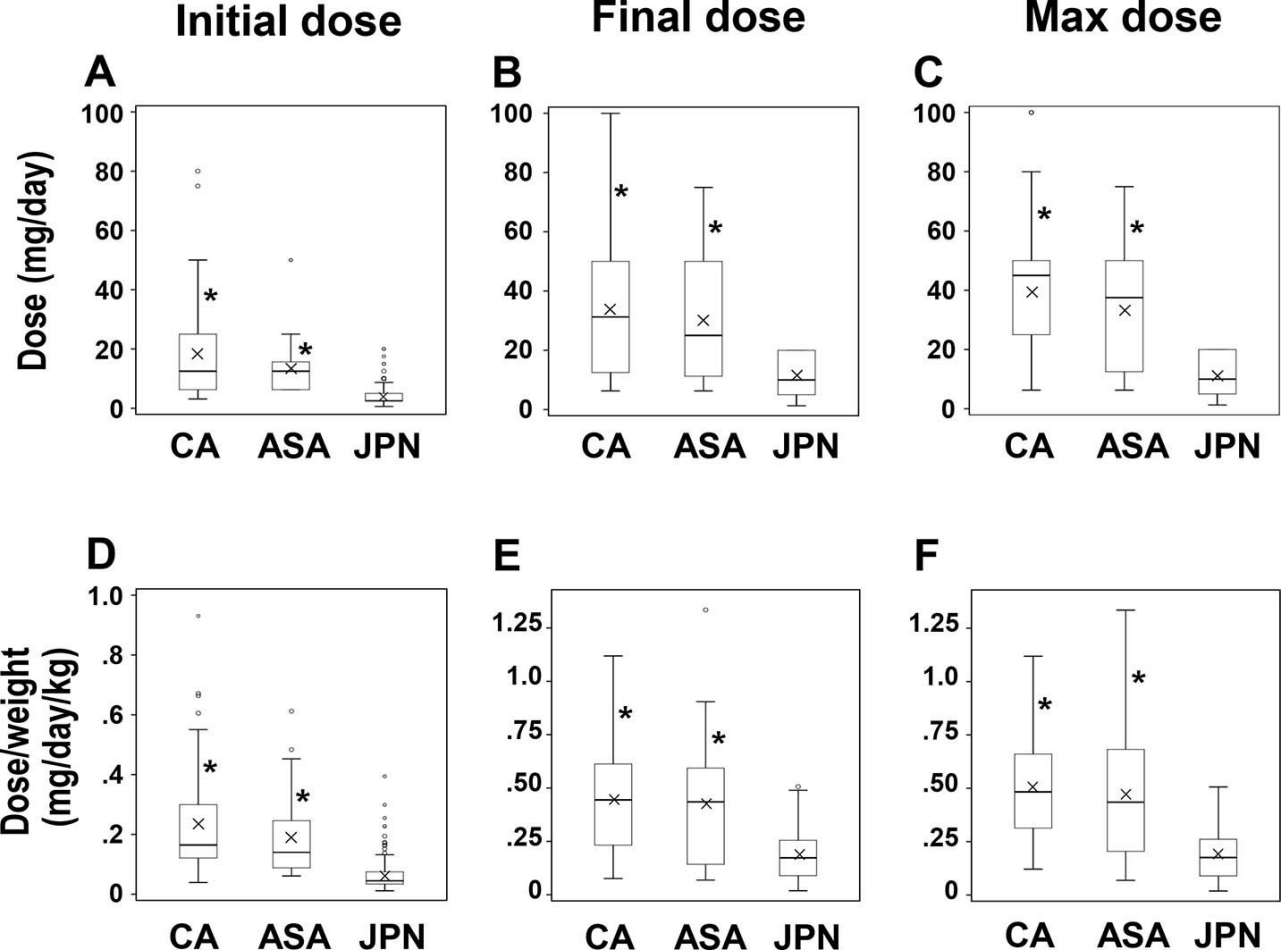
Dose difference of carvedilol among Caucasians, Asian Americans, and Japanese. (a), (b) and (c) indicate the box-and-whisker plots for the Initial dose (mg/day), Final dose (mg/day), and Maximal dose (mg/day); (d), Initial dose per weight (mg/day/kg); (e), Final dose per weight (mg/day/kg); (f), Maximal dose per weight (mg/day/kg); *, *p* < 0.001 compared with Japanese by Kruskal-Wallis and Wilcoxon rank-sum test; CA, Caucasian (n = 38); ASA, Asian American (n = 28); JPN, Japanese (n = 93); x in each box indicates the mean value; n, number of patients.

Fig 2 shows the differences in the carvedilol doses for the initial (a), final (b), and maximal (c) among the three groups. JPNs had significantly lower initial, final, and maximal doses than CAs or ASAs (*p* < 0.001, Wilcoxon rank-sum test). When adjusted for weight (mg/day/kg), the initial (d), final (e), and maximal (f) carvedilol doses remained significantly lower than CAs or ASAs (*p* < 0.001, Wilcoxon rank-sum test). Details of the values constituting Fig 2 can be found in the supplementary materials (S3 Table). JPNs also demonstrated significantly lower doses than USPs (n = 66 for USPs, initial, 17.1 ± 16.2 mg/day; final, 33.0 ± 21.8 mg/day; maximal, 36.8 ± 22.8 mg/day, *p* < 0.001, Wilcoxon rank-sum test, respectively).

As shown in Table 3, 39.5% of CAs, 35.7% of ASAs, and 25.8% of JPNs were titrated to achieve the target dose of carvedilol in each national GDMT (50 mg/day for CAs and ASAs, 20 mg for JPNs), and almost all of the study population was treated with or within each target dose (94.8% of CAs, 92.9% of ASAs, 100% of JPNs). The frequency of USPs treated less than their national GDMT target dose of 50 mg/day was 56.1%. On the other hand, 74.2% of JPNs were treated with less than the Japanese GDMT target dose of 20 mg/day, and it was significantly higher than that of USPs with a relative rate of 1.32 (95% CI: 1.04 to 1.69, *p* = 0.026).

**Table 3:** Rates of dose achievement in GDMT.

Race (N) Final dose	Caucasian (38)	Asian American (28)	Japanese (93)	*p-v*alue
**<Target dose**	55.3% (21)	57.1% (16)	74.2% (69)	0.022
**= Target dose**	39.5% (15)	35.7% (10)	25.8% (24)	
**>50 mg/day**	5.2% (2)	7.1% (2)	0.0% (0)	

GDMT: guideline-directed medical therapy; Target dose: target dose of carvedilol in each GDMT, 50 mg/day for Caucasian and Asian Americans, 20 mg/day for Japanese.

The parentheses indicate the patients who achieved less than the target dose, *p*-value: by 3x3 Fisher’s exact test.

### Clinical outcomes of carvedilol

**Fig 3:**
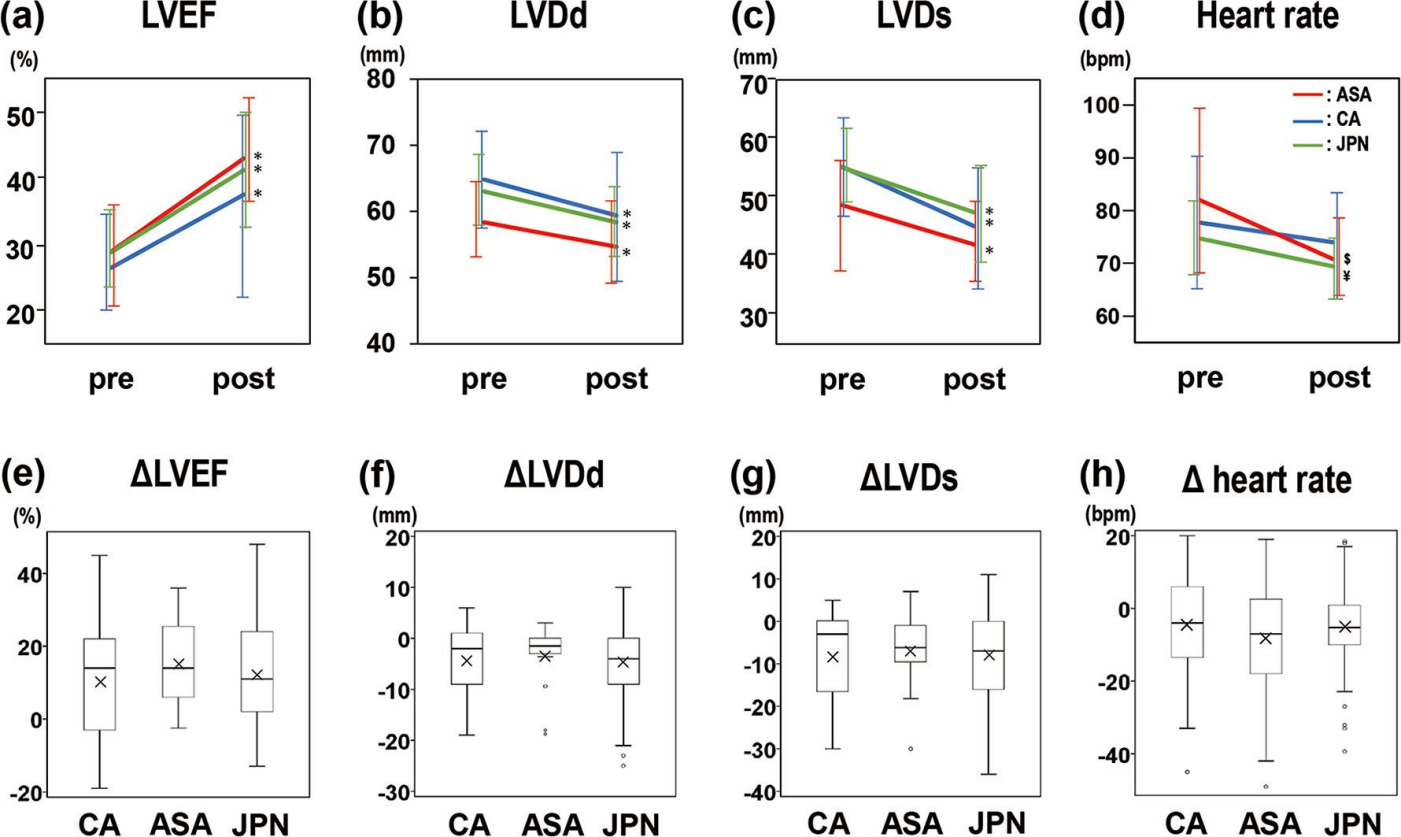
Comparison of carvedilol effects on the left ventricle and heart rate among groups. (a), (b), (c), and (d) indicate time-dependent changes in LVEF (%), LVDd (mm), LVDs (mm), and heart rate (bpm); Blue line for Caucasians, Red line for Asian Americans, Green line for Japanese; pre, pre-treatment with carvedilol; post, post-treatment with carvedilol; * in (a), (b), and (c), *p* < 0.05 compared with pre-treatment for each race by t-test; $ in (d), *p* < 0.05 compared to the pre-treatment for Asian Americans by paired t-test; ¥ in (d), *p* < 0.05 compared to the pre-treatment for Japanese by paired t-test. (e), (f), (g), and (h) indicate the box-and-whisker plots for the absolute change between pre-treatments and post-treatments with carvedilol in LVEF (%), LVDd (mm), LVDs (mm), and heart rate (bpm); Δ, absolute change; CA, Caucasian; ASA, Asian American; JPN, Japanese; n, number of subjects. X in each box indicates the mean value.

As shown in Fig 3, LVEF (a), LVDd (b), and LVDs (c) changed significantly after carvedilol treatment was initiated in each group; heart rate (d) decreased in all groups after the treatment, with a significant decrease found only in ASAs and JPNs, but not in CAs (*p* < 0.05, pre vs. post paired t-test in each group). Changes in LVEF, LVDd, LVDs, and heart rate after treatment with carvedilol were evaluated and denoted as ΔLVEF (e), ΔLVDd (f), ΔLVDs (g), and Δheart rate (h). The figures show no notable difference in each parameter among the races, even with a significant difference in carvedilol dosing. The mean differences with [95% CI] between JPNs and USPs were −0.68 [−5.49 to 4.12] for ΔLVEF (%), −0.55 [−3.24 to 2.15] for ΔLVDd (mm), −0.25 [−1.92 to 1.43] for ΔLVDd index (mm/BSA), 0.03 [−3.84 to 3.90] for ΔLVDs (mm), −0.04 [−2.38 to 2.30] for ΔLVDs index (mm/BSA), and 1.62 [−3.07 to 6.32] for Δheart rate (bpm). Each mean difference was slight, and its 95% CI almost evenly straddled zero. Details of each value constituting Fig 3 are in the supplemental materials (S4 Table).

The rates of the patients whose LVEF recovered to more than 40% one year after the carvedilol treatment were comparable among the three groups (CAs, 41.4%; ASAs, 47.8%; JPNs, 48.8%, *p* = 0.739, 2x3 Chi-square test).

**Fig 4:**
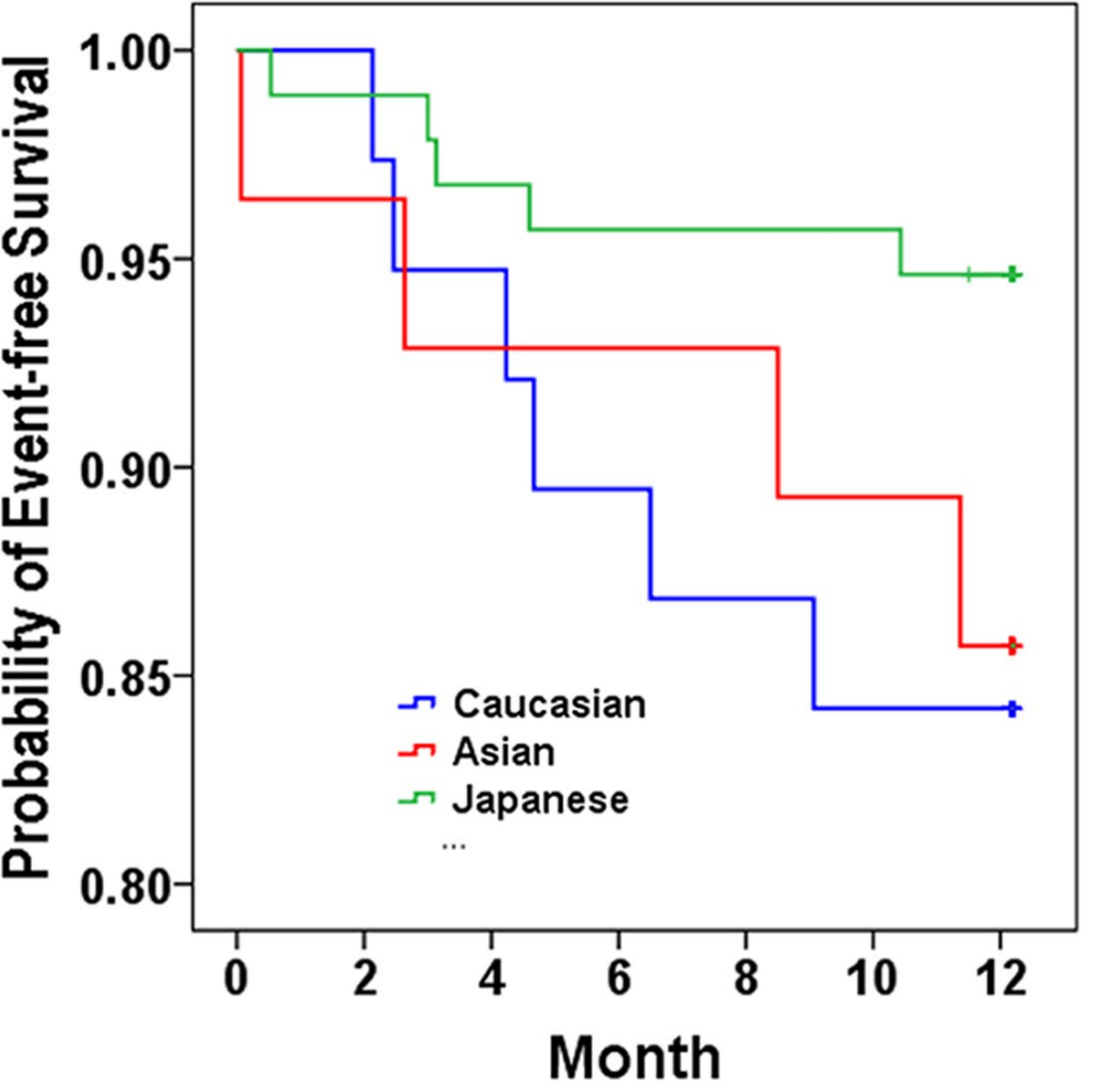
Kaplan-Meier plot of event-free estimate during a one-year follow-up period. Kaplan–Meier analysis shows the probability of patients without CVD hospitalization or death during one-year follow-up after carvedilol initiation. Blue line for Caucasians, Red line for Asian Americans, Green line for Japanese. *p* = 0.172 by log-rank test.

Finally, Fig 4 shows a Kaplan-Meier plot of the event-free survival one year after the initiation of the carvedilol. The probability of event-free survival showed a lower trend in the CAs; however, this did not reach statistical significance among the three groups (*p* = 0.172, log-rank test). The details of the events are summarized in S5 Table.

## Discussion

In this study, we observed that race might play a role in the optimal dose of carvedilol for HFrEF management by comparing the dose of carvedilol between JPNs and USPs, composed CAs and ASAs, with HFrEF and the effects of different carvedilol doses on heart functions and tolerability using real-world data from this international collaborative clinical study. The GDMT sequence with ß-blockers to manage HFrEF patients remains similar to the guidelines between the US and Japan except for the initial and target doses. As a result, HFrEF patients in the US received more than twice the initial, final, and maximum doses of carvedilol compared to patients in Japan. JPNs had a higher rate of patients treated with less than the national GDMT target dose than USPs. However, the frequencies of patients whose LVEF recovered by more than 40% were comparable among the races, even though JPNs exhibited markedly lower doses than USPs.

Furthermore, the mean differences of changes in cardiac functional parameters between the USPs and JPNs were minimal, and the 95% CI for each parameter was within a narrow range and almost evenly straddled zero. These results suggest that the examined parameters might demonstrate the non-inferiority of JPNs to USPs. For example, a study quantitatively assessed Δheart rate using the subjects with LVEF< 45% and 20 mg/day of carvedilol treatment and showed no statistically significant difference in Δheart rate despite the mean difference of 15.1 bpm [14]. Referring to the data and assuming the non-inferiority margin of 7.5 bpm for Δheart rate, we postulated that JPNs could show non-inferiority in Δheart rate compared to USPs by the evidence that the mean difference of 1.62 was within the margin and the upper limit of 95% CI (-3.07 to 6.32) did not exceed the threshold for the non-inferiority margin.

Genetic and environmental factors are essential variables that determine the pharmacokinetics (PK) and pharmacodynamics (PD), directly impacting drug efficacy and safety. The PK of carvedilol is known to exhibit a four- to five-fold interindividual variability due to genetic and environmental factors [15, 16]; however, the PK trials of carvedilol with Japanese and Caucasian healthy volunteers living in Japan by the pharmaceutical company concluded that there was no significant difference between them [17]. Because carvedilol is metabolized by multiple cytochromes P450 (CYP) (CYP2D6, CYP2C19, CYP3A4, CYP2E1, and CYP1A2) and transported by proteins, such as ABCB1 [18], the clinical impact of individual genetic polymorphisms may be challenging to confirm. In this study, JPNs displayed a smaller LVEF improvement than ASAs but showed a more LVEF improvement than CAs, even though JPNs received significantly lower doses of carvedilol than those in CAs. Regarding heart rate, the degree of heart rate reduction by β-blockers was reported to depend on the baseline heart rate [19]. While there was no significant difference in the baseline heart rate among the races, both ASAs and JPNs demonstrated a significant decrease in heart rate, respectively, but CAs did not (as per the paired t-test) despite having a slightly higher baseline heart rate and receiving a significantly higher dose than JPNs. This might be explained by genetic variants related to β-receptor activity, such as one variant of ADRB2 Glu27Gln, which showed a greater reduction in blood pressure with carvedilol compared to the variant ADRB2 Glu27Glu [20] because the frequencies of the G allele, which is in Glu, are 42.4% for Caucasian and 14.3% for Asian [21]. Considering carvedilol’s negative cardiac ionotropic and chronotropic effects, Asians might have shown higher pharmacological responsiveness to carvedilol than CAs. The rate of carvedilol discontinuation indicates that ASAs with high-dose treatment might tend to be the least tolerable to carvedilol among the three groups. Generally, the responsiveness of the dominant race enrolled in the clinical trials governs the results, which are used as a reference to set up the guidelines. As the Asian population seems to be enrolled rarely in clinical trials conducted in the US or the EU countries [22, 23], the trials’ results may not accurately reflect the PK and PD of carvedilol in the Asian American population, and this is intimately related to drug effectiveness, tolerability, and safety. Future studies would be needed to investigate the roles of gene-gene and gene-environment interactions on the clinical impact of carvedilol for HFrEF management. However, our observational study proposes that the US-recommended dose of carvedilol might be too high for some ASAs, and careful ß-blocker-dose initiation and titration should be implemented with these results in mind during HFrEF management.

As with other observational studies, our findings should be interpreted in the context of several potentially important limitations. First, the sample size was too small, especially for ASAs and CAs, to test the clinical effectiveness of different dosages sufficiently. The primary reason for the small sample size was resource and time constraints for selecting additional study institutions. The secondary reason is that the documentation of the US patients by self-reported ethnicity or race was very difficult. Therefore, the patients with unclarified race were excluded, resulting in a small number of subjects. Another reason for the small sample size is that our inclusion and exclusion criteria, based on the pivotal RCTs [10, 12] performed previously in the US and Japan, may have been too restrictive. However, more than 26 subjects per group could maintain statistical power of more than 90% to analyze dose differences. Second, the categorization of ASAs and JPNs included in this study and considered “Asian” may include a more heterogeneous group than we assume. Initially, we planned to compare the difference in carvedilol doses and the effects of the different doses between Japanese and Japanese Americans treated according to their national GDMT, respectively. However, we found it challenging to collect enough Japanese Americans exclusively. Then, ASAs were selected for comparison to Japanese, although genetic diversity exists even among the same regional population of Asia [24]. We expect that additional clinical studies with larger sample sizes and more specified races would help to verify the results, especially clinical outcomes. Third, although there was no difference in event-free survival during the one-year follow-up period among the groups, we could not adjust the difference in the medical care system or follow-up system after the initiation of β-blocker between the US and Japan to compare the effectiveness. Fourth, there was a discrepancy in the study periods between the US and Japan because the electronic data extraction processes in the US were established earlier than in Japan, allowing us to access a broader range of historical data. While we acknowledge that the difference in data collection periods between the institutions might affect the comparability of our findings, we believe that the insights provided by our study still offer valuable contributions to the field. Future research could aim to align data collection periods more closely, which would further validate and expand upon our findings. Lastly, as this study was performed before JCS proposed the new HF guideline in 2021 [4] and AHA/ACC/HFSA in 2022 [3], we did not consider the effects of SGLT2 inhibitors and ARNI as concomitant medications in this study. However, we propose that a lower dose of carvedilol could suffice for HFrEF management with concomitant GDMT in some Asian populations with baseline demographics and HFrEF status similar to those of this study’s subjects.

## Conclusions

In conclusion, HFrEF patients from the two regions (treated as per their national guideline recommendations) showed significant differences in carvedilol doses. Evaluating the effects of different carvedilol doses revealed that JPNs demonstrated the possibility of non-inferior effectiveness and preferable tolerability compared to USPs combined CAs and ASAs despite receiving markedly lower doses. These differences may be attributed to genetic polymorphisms in the genes related to the responsiveness of β-adrenergic blockers in Asian populations that warrant lower doses compared to CAs.

We suggest that some Asian populations with HFrEF could sufficiently respond to lower doses of carvedilol than those of GDMT recommended by ACC/AHA and ESC., and dose de-escalation, not discontinuation, may be an option for some Asian and ASA HFrEF patients intolerable to high doses of carvedilol.

## Supporting information

S1 Table The recommended daily dose of ß-blockers in GDMT for HFrEF. (DOCX)

S2 Table Demographics and baseline characteristics of US patients (USPs). (DOCX)

S3 Table Dose (mg/day) and dose/weight (mg/day/kg) of each group. (DOCX)

S4 Table Data related to heart function. (DOCX)

S5 Table Number of patients and details of the events. (DOCX)
