# Supplementary material for: Lower doses of carvedilol in Japanese heart failure patients with reduced ejection fraction could show the potential to be non-inferior to higher doses in US patients: An international collaborative observational study
Source: PLoS One. 2024 Mar 7;19(3):e0299510. doi: 10.1371/journal.pone.0299510 (PMC10919845; doi:10.1371/journal.pone.0299510)
Supplement: S1 Table — (DOCX) [file pone.0299510.s001.docx]

**S1 Table. The recommended daily dose of ß-blockers in GDMT for HFrEF**

| **Drug**  **(times/day)** | **Carvedilol**  **(2)** | | **Bisoprolol**  **(1)** | | **Metoprolol CR/XL (1)** | |
| --- | --- | --- | --- | --- | --- | --- |
| **Dose (mg/day)**  **Organizer**  **of guideline** | **Initial** | **Target** | **Initial** | **Target** | **Initial** | **Target** |
| **ACC/AHA** | 6.25 | 50–100 | 1.25 | 10 | 12.5–25 | 200 |
| **JCS** | 2.5 | 20 | 0.625 | 5 | not approved | |

GDMT: guideline-directed medical therapy; ACC: American College of Cardiology; AHA: American Heart Association; JCS: Japanese Circulation Society
