# Supplementary material for: Lower doses of carvedilol in Japanese heart failure patients with reduced ejection fraction could show the potential to be non-inferior to higher doses in US patients: An international collaborative observational study
Source: PLoS One. 2024 Mar 7;19(3):e0299510. doi: 10.1371/journal.pone.0299510 (PMC10919845; doi:10.1371/journal.pone.0299510)
Supplement: S2 Table — (DOCX) [file pone.0299510.s002.docx]

**S2 Table. Demographics and baseline characteristics of US patients (USPs)**

|  | US Patients (n = 66) | *p*-value vs JPNs |
| --- | --- | --- |
| Age (years) | 58.8 (17.6)  58.5 (48.8–73.3)  66 | 0.474 |
| Weight (kg) | 76.1 (19.2) †  75.7 (62.5–87.5)  66 | <0.001 |
| Height (cm) | 169.2 (9.5) *  170.2 (162.6–177.8)  62 | 0.002 |
| BMI (kg/m^2^) | 26.4 (5.1) *  25.7 (22.9–29.4)  62 | <0.001 |
| Sex | | |
| Man | 45 (68.2) | 0.818 |
| Women | 21 (31.8) |  |
| Smoking | | |
| Never | 33 (55.0) | 0.803 |
| Current | 10 (16.7) |  |
| Past | 17 (28.3) |  |
| NYHA class | | |
| Ⅰ | 6 (15.4) | 0.846 |
| Ⅱ/Ⅲ | 32 (82.0) |  |
| Ⅳ | 1 (2.6) |  |
| Heart rhythm | | |
| Sinus | 53 (84.1) | 0.124 |
| AF | 10 (15.9) |  |
| SBP (mmHg) | 121.6 (20.0)  120.0 (108.0–137.0)  59 | 0.181 |
| DBP (mmHg) | 73.8 (14.8)  73.0 (66.0–81.0)  59 | 0.184 |
| eGFR (ml/min/1.73m^2^) | | |
| >50 | 37 (88.1) | 0.165 |
| ≤50 | 5 (11.9) |  |
| Medications | | |
| ACEi / ARB | 53 (80.3) | 0.957 |
| Diuretics | 46 (69.7) | 0.794 |
| MRA | 23 (34.8) | 0.627 |
| Digoxin | 13 (65.0) | 0.023 |
