# Supplementary material for: Lower doses of carvedilol in Japanese heart failure patients with reduced ejection fraction could show the potential to be non-inferior to higher doses in US patients: An international collaborative observational study
Source: PLoS One. 2024 Mar 7;19(3):e0299510. doi: 10.1371/journal.pone.0299510 (PMC10919845; doi:10.1371/journal.pone.0299510)
Supplement: S3 Table — (DOCX) [file pone.0299510.s003.docx]

**S3 Table. Dose (mg/day) and dose/weight (mg/day/kg) of each group**

|  | **Caucasian (38)** | | **Asian (28)** | | **Japanese (93)** | **p-value** |
| --- | --- | --- | --- | --- | --- | --- |
| **Dose (mg/day)** | | | | | |  |
| **Initial** | 19.1 (18.7)*  12.5 (6.3–25.0)  38 | 14.5 (11.9)*  12.5 (6.3–17.2)  28 | | 4.3 (4.2)  2.5 (2.5–5.0)  93 | | <0.001 |
| **Max** | 39.2 (23.5)*  45.0 (21.9–50.0)  38 | 33.6 (21.8)*  37.5 (12.5–50.0)  28 | | 11.3 (6.5)  10.0 (5.0–20.0)  93 | | <0.001 |
| **Final** | 34.5 (21.4)*  31.3 (12.5–50.0)  38 | 30.9 (22.4)*  25.0 (10.6–50.0)  28 | | 11.2 (6.5)  10.0 (5.0–20.0)  93 | | <0.001 |
| **Dose/weight (mg/day/kg)** | | | | | |  |
| **Initial** | 0.24 (0.20) *  0.16 (0.11–0.31)  38 | 0.20 (0.15) *  0.14 (0.09–0.25)  28 | | 0.07 (0.07)  0.04 (0.03–0.08)  93 | | <0.001 |
| **Max** | 0.50 (0.25) *  0.48 (0.30–0.66)  38 | 0.46 (0.32) *0.43 (0.20–0.70)  28 | | 0.19 (0.11)  0.18 (0.09–0.26)  93 | | <0.001 |
| **Final** | 0.44 (0.26) *  0.44 (0.22–0.62)  38 | 0.42 (0.31) *  0.43 (0.14–0.60)  28 | | 0.19 (0.11)  0.17 (0.09–0.26)  93 | | <0.001 |

The top shows the mean with the SD in brackets, the middle shows the median with the IQR in brackets, and the bottom shows the number of patients. *, p< 0.001 compared with Japanese by Kruskal-Wallis and Wilcoxon rank-sum test.
