# Supplementary material for: Lower doses of carvedilol in Japanese heart failure patients with reduced ejection fraction could show the potential to be non-inferior to higher doses in US patients: An international collaborative observational study
Source: PLoS One. 2024 Mar 7;19(3):e0299510. doi: 10.1371/journal.pone.0299510 (PMC10919845; doi:10.1371/journal.pone.0299510)
Supplement: S4 Table — (DOCX) [file pone.0299510.s004.docx]

**S4 Table. Data related to heart function**

|  | **Caucasian (38)** | **Asian (28)** | **US patients (66)** | **Japanese (93)** |
| --- | --- | --- | --- | --- |
| **LVEF (%)** | | | |  |
| **Initial** | 26.5 (8.1)  24.5 (19.8–34.0)  38 | 28.9 (8.8)  31.0 (20.5–36.0)  28 | 27.5 (8.4)  28.0 (20.0–36.0)  66 | 28.8 (7.4)  30.0 (23.0–35.0)  93 |
| **Final** | 37.5 (15.3)  35.5 (21.8–49.0)  30 | 42.9 (11.1)  42.0 (36.5–51.0)  25 | 40.0 (13.7)  40.0 (30.0–49.0)  55 | 41.2 (13.1)  40.0 (32.0–49.5)  85 |
| **Absolute change** | 11.9 (15.3)  14.0 (−3.0–22.8)  30 | 15.2 (12.1)  14.0 (4.0–26.3)  25 | 13.4 (13.9)  14.0 (0.0–25.0)  55 | 12.7 (14.5)  11.0 (2.0–24.5)  85 |
| **LVDd (mm)** | | | |  |
| **Initial** | 64.8 (14.0)  64.0 (57.8–72.3)  30 | 58.4 (7.8)  56.0 (53.0–64.5)  21 | 62.2 (12.1)  63.0 (54.0–69.0)  51 | 63.0 (8.6)  62.0 (58.0–68.0)  93 |
| **Final** | 59.4 (14.1)  55.0 (49.6–68.5)  25 | 54.7 (9.1)  54.5 (49.0–61.6)  21 | 57.2 (12.2)  54.8 (49.8–65.3)  46 | 58.4 (9.9)  57.0 (53.0–63.0)  91 |
| **Absolute change** | −4.7 (7.9)  −2.0 (−9.0–1.0)  21 | −3.4 (6.2)  −1.5 (−3.3–0.0)  17 | −4.1 (7.1)  −2.0 (−9.0−0.1)  38 | −4.7 (7.1)  −4.0 (−9.0–0.3)  91 |
| **LVDd index (mm/m^2^)** | | | |  |
| **Initial** | 34.3 (5.4)  33.5 (30.5–38.8)  26 | 33.8 (4.9)  34.0 (30.9–38.2)  21 | 34.1 (5.1)  34.0 (30.7–38.4)  47 | 38.3 (5.7)  37.9 (34.1–41.7)  93 |
| **Final** | 31.4 (5.2)  30.4 (26.5–34.9)  22 | 30.1 (5.0)  29.6 (26.4–34.5)  21 | 30.8 (5.1)  29.8 (26.6–34.5)  43 | 35.5 (6.8)  35.1 (29.6–39.7)  91 |
| **Absolute change** | −2.8 (4.4)  −1.1 (−5.3–0.4)  19 | −2.1 (4.1)  −1.0 (−1.7–0.0)  17 | –2.4 (4.2)  –1.0 (–4.7–0.0)  36 | −2.8 (4.3)  −2.1 (−5.5–0.0)  91 |
| **LVDs (mm)** | | | |  |
| **Initial** | 55.2 (14.4)  54.5 (46.0–63.3)  30 | 48.7 (11.1)  49.0 (37.8–56.5)  20 | 52.6 (13.5)  52.5 (43.0–61.3)  50 | 55.1 (9.5)  54.0 (49.0–61.0)  93 |
| **Final** | 45.1 (16.9)  40.0 (33.2–54.2)  25 | 42.0 (10.6)  42.2 (34.8–49.2)  21 | 43.7 (14.3)  41.8 (33.8−51.4)  46 | 47.4 (12.0)  45.0 (38.8–55.0)  90 |
| **Absolute change** | −8.9 (11.3)  −4.0 (−18.5–0.2)  20 | −6.6 (8.7)  −6.2 (−9.8–0.9)  16 | −7.9 (10.2)  −5.4 (−15.3– −0.23)  36 | −7.8 (9.6)  −7.0 (−16.0–0.0)  90 |
| **LVDs index (mm/m^2^)** | | | |  |
| **Initial** | 29.4 (5.8)  28.4 (25.2–33.9)  26 | 27.8 (5.4)  27.4 (25.0–32.8)  20 | 28.7 (5.6)  28.2 (25.2–33.3)  46 | 33.4 (5.9)  32.6 (28.4–37.8)  93 |
| **Final** | 23.8 (7.1)  21.9 (18.0–27.9)  22 | 23.0 (5.6)  23.2 (18.3–27.4)  21 | 23.4 (6.4)  22.6 (18.2–27.8)  43 | 28.9 (7.8)  28.2 (23.5–33.7)  90 |
| **Absolute change** | −5.2 (6.5)  −2.6 (−12.3–0.1)  17 | −3.8 (5.4)  −3.6 (−5.1– −0.5)  16 | –4.6 (5.9)  –3.2 (−8.6– −0.2)  33 | −4.6 (5.6)  −4.1 (−9.3–0.0)  90− |
| **Heart rate (beats per min)** | | | |  |
| **Initial** | 77.7 (13.5)  76.0 (65.8–90.5)  34 | 81.9 (19.0) 77.0 (68.0–99.0)  27 | 79.5 (16.2)  76.0 (66.5–93.0)  61 | 74.7 (10.9)  73.0 (67.7–81.7)  93 |
| **Final** | 74.0 (12.5)  74.5 (62.3–83.0)  34 | 70.8 (10.6)  70.0 (63.3–78.8)  24 | 72.7 (11.8)  71.5 (63.0–82.0)  58 | 69.4 (9.4)  69.8 (62.3–75.0)  93 |
| **Absolute change** | −4.8 (14.5)  −4.0 (−13.8–7.0)  32 | −9.6 (17.6)  −7.0 (−18.0–3.3)  24 | −6.9 (16.0)  –4.5 (–15.0–6.3)  56 | −5.2 (10.4)  −5.3 (−10.0–1.0)  93 |

The Top in the box; the mean with the SD, the middle; the median with the IQR, the bottom; the number of patients, the fourth; the difference from Japanese with 95% CI. Absolute change: change amount from the baseline.
