# Supplementary material for: Lower doses of carvedilol in Japanese heart failure patients with reduced ejection fraction could show the potential to be non-inferior to higher doses in US patients: An international collaborative observational study
Source: PLoS One. 2024 Mar 7;19(3):e0299510. doi: 10.1371/journal.pone.0299510 (PMC10919845; doi:10.1371/journal.pone.0299510)
Supplement: S5 Table — (DOCX) [file pone.0299510.s005.docx]

**S5 Table. Number of patients and details of the events**

| **Race (n)** | **Caucasian (38)** | **Asian American (28)** | **Japanese (93)** |
| --- | --- | --- | --- |
| **Hospitalization for cardiac events** | 6;  Sob (1),  Possible stroke (1),  NSTEMI (1),  CHF exacerbation (3) | 4;  Chest pain (1),  CHF exacerbation (3) | 4;  CHF exacerbation (2),  Tachyarrhythmia (1),  Dizziness (1) |
| **Death** | 0 | 0 | 1;  due to interstitial pneumonia |
